# The S. S. White Electric Furnace “B” with Pyrometer

**Published:** 1923-03

**Authors:** 


### THE S. S. WHITE ELECTRIC FURNACE "B" WITH PYROMETER

### A VASTLY IMPROVED EQUIPMENT FOR THE "FIRING" OF DENTAL PORCELAIN

Recognizing the need for more accuracy and greater permanence in the temperature indications in porcelain firing, we have introduced a new Pyrometer Element in conjunction with the well known S. S. White Electric Furnace by which the uncertainties due to inherent deficiencies in the Indicator and Thermo-Couple formerly used, are now eliminated.

It would be unfair to the former equipment to simply cast it aside as a wholly unsatisfactory organization, for in its time and with regard for the stage of development to which Pyrometers generally had advanced, the S. S. White Pyrometer Furnace "A" was a creditable achievement and has served a useful purpose in the advancement of the art of porcelain restorations in dentistry.

What has actually happened is this—a more accurate, more dependable and more practical type of indicator or milli-volt meter has been developed enabling us to use a better and more constant thermo-couple than could be employed with any former commercial type of indicator. Both the indicator and the couple in Furnace "B" are of a higher scientific standard and of a laboratory accuracy previously impossible with the commercial types of elements we were compelled to employ.

The result of the improvements not only affects the character of the porcelain work and the facility with which it can be done; it also affects the life of the muffle in minimizing the liability to overheating, by providing permanently accurate indications of the muffle temperature.

It might be explained that the combination of metals (platinum and platinum alloy) in the thermo-couple previously used, while not affording the desirable constancy of the new combination, were adopted because they produced a greater thermo potential necessary for visible indications in the best type of instrument or indicator then commercially available.

The new instrument has a higher sensibility—is more responsive to delicate changes in the e. m. f. generated by the thermo-couple, than the instrument formerly employed, yet it has the ruggedness to withstand shipment and continuous use without deterioration of its calibration or detriment of its delicate mechanism.

It is somewhat difficult to picture the exact requirements of a perfect commercial or transportable type of pyrometer without entering into a thorough analysis of thermoelectrics and of milli-volt meter construction and dynamics. The facts, however, are available to any one sufficiently interested to read the scientific papers on the subject and we will be glad to give more detail to those who want it.

Briefly we may state the advantages of the S. S. White Pyrometer Furnace "B" as follows:

1. The same general excellence of the muffle, resistance, heat control and mechanical construction.

2. A higher degree of accuracy in the indicator.

3. Constancy of calibration and reliable operation of the indicator.

4. Greater visibility of the scale indications due to the new position of the instrument.

5. Vertical support of the pivots on the moving element in the indicator avoiding fractional retardation of the pointer or indicating needle.

6. A Platinum and Platinum-Rhodium thermo-couple of high constancy, approved by authorities on pyrometry.

7. Better spacing of the couple wires in the muffle.

8. Greater distance of the "cold-junction" from the heat of the muffle, a distinct advantage in the non-interference with the accuracy by stray heat currents and thermal changes outside the muffle.

9. Better protection and support for the connecting leads from the thermo-couple to the indicator.

10. A more practical, more reliable, more accurate Dental Pyrometer Furnace than has ever been offered to the profession.

This last statement is somewhat bolder than we usually make, concerning our manufactures but the market for dental furnaces has not been such as to invite the expenditure of money and effort for development by many producers. We believe that we have done more work along this line than any or all other manufacturers and our work has been done in the confidence that porcelain work in dentistry is here to stay and to grow in importance.

As a matter of fact the sales of our furnaces have grown from a low minimum to which they fell three years ago, to a very satisfactory output in the last year and we have confidence in the increasing use of them especially in view of the splendid improvements we are able to offer.

The introduction of the new type of instrument and the improvements in the couple construction have necessitated a higher cost, so that in spite of our desire to lower the price of the equipment or even to hold it at the former level we are required to get more money for the pyrometer than we formerly charged.

The prices for muffles we have reduced somewhat on account of the general reduction in the cost of platinum, but the mechanical costs are higher and the new instrument costs much more.

However, we believe that porcelain workers realize the desirability of the highest grade equipment and that they will willingly pay more for better service.

